# Swine inflammation and necrosis syndrome is influenced by husbandry and quality of sow in suckling piglets, weaners and fattening pigs

**DOI:** 10.1186/s40813-020-00170-2

**Published:** 2020-11-23

**Authors:** Gerald Reiner, Josef Kühling, Mirjam Lechner, Hansjörg Schrade, Janine Saltzmann, Christoph Muelling, Sven Dänicke, Frederik Loewenstein

**Affiliations:** 1 Department of Veterinary Clinical Sciences, Clinic for Swine, Justus-Liebig-University, Frankfurter Strasse 112, 35392 Giessen, Germany; 2 UEG Hohenlohe, Am Wasen 20, 91567 Herrieden, Germany; 3 LSZ Boxberg, Seehöfer Str. 50, 97944 Boxberg, Germany; 4 Institute of Animal Nutrition, Friedrich-Loeffler-Institut, Federal Research Institute for Animal Health, Bundesallee 37, 38116 Braunschweig, Germany; 5 Institute for Veterinary Anatomy, University Leipzig, An den Tierkliniken 43, 04103 Leipzig, Germany; 6 Institute of Veterinary Pathology, University Leipzig, An den Tierkliniken 33, 04103 Leipzig, Germany

**Keywords:** Swine inflammation and necrosis, Clinical study, Histopathology, Coprostasis, Animal welfare

## Abstract

**Background:**

Swine inflammation and necrosis syndrome (SINS) is a newly identified syndrome in swine that can affect different parts of the extremities in suckling piglets. This study investigates the hypotheses that the clinical signs of SINS have histological equivalents, that SINS can also be observed in weaners and fatteners, that improving sow quality and husbandry (here the supply of water and fibre) can reduce the signs, and that coprostasis in sows is significantly associated with SINS in their offspring.

From a cohort of 123 hybrid sows, the twenty sows exhibiting the best conditions and the twenty exhibiting the worst conditions were selected based on detailed scores from coronary bands, soles, heels, claws and teats. Half of the sows in each group, along with their offspring, were kept under conventional conditions, while the environment for the remaining sows in each group was improved with drinking bowls, water disinfection and additional feeding with hay and straw. In total, 115 suckling piglets, 113 weaners and 103 fatteners were scored for the degree of inflammation and necrosis of their tails, ears, teats, coronary bands, soles, heels and claws.

**Results:**

The clinical signs of SINS are associated with inflammatory signs at the histological level. SINS scores in suckling piglets, weaners and fatteners derived from low-quality sows under standard husbandry conditions were high, but they decreased significantly when husbandry was improved (water consumption and additional fibre). Sow quality had significant effects on suckling piglets and weaners under standard husbandry conditions. Coprostasis in sows led to significantly higher SINS scores in their offspring at any age. Improved husbandry conditions were associated with a reduced prevalence of coprostasis (R^2^ = 0.74). Taking all factors together, husbandry improvements, sow quality and coprostasis explained 57, 67 and 45% of SINS score variance in suckling piglets, weaners and fatteners, respectively.

**Conclusion:**

The present study shows that SINS is not limited to suckling piglets but can also be found in weaners and fatteners. Coprostasis in sows is significantly correlated with SINS in their offspring and adds a good prognostic tool. Water supply and fibre could play a crucial role in combatting the syndrome.

## Background

Swine inflammation and necrosis syndrome (SINS) is a newly identified syndrome that leads to significant clinical findings of inflammation and necrosis in the extremities in pigs [29, 50]. Any signs of inflammation and loss of tail integrity indicate serious impairment of animal welfare [14, 15], the preservation of which is one of the major challenges facing modern pig farming. Thus, there is an urgent need to raise awareness among pig farmers and to collect information that can help counteract the syndrome.

Tail biting due to behavioural factors is a major cause of considerable wellbeing reduction in pigs [58–60], but tail lesions can also arise without action from other penmates [2, 29, 30, 43, 50–52]. Ears are a second organ complex repeatedly associated with inflammation and necrosis [37, 38, 42, 47, 66]. Often there is little clarity about the triggers. Pathogens such as treponema, staphylococci or mycoplasma have been discussed [37, 42, 47], but it remains unclear whether the pathogens were the cause or result of the lesions. Weissenbacher-Lang et al. [66] found no histological evidence for the primary involvement of pathogens and suspected non-infectious causes such as mycotoxins and stress as an aetiology.

Our research on SINS has shown that piglets with inflammation and necrosis around tails and ears may be affected in even more organ systems, in particular the heels, claw coronary bands, teats, umbilicus, vulva and face [50, 51].

The first hypothesis tested in this study was whether the clinical signs were associated with inflammation at the histological level. Since more and more evidence is emerging in the field that SINS also occurs in weaners and fattening pigs [50], the second hypothesis was that SINS can also occur after weaning.

Due to the diversity of the organ systems involved and because clinical inflammation and necrosis can occur within the first days of life [51], the probability that biting or technopathies such as unfavourable floor conditions are the sole aetiologies is reduced, even if such effects are likely to play a decisive role in the final expression of the signs. However, several body parts might be affected simultaneously due to the transfer of bacterial degradation products (e.g., LPS [lipopolysaccharides]) and mycotoxins (e.g., DON [deoxynivalenol]) into the bloodstream, and LPS and mycotoxins from sow’s milk might be directly associated with necrosis of tails, ears and coronary bands in suckling piglets [8, 18, 23, 50, 51, 54, 63, 66]. The central point of attack for mycotoxins and particularly for DON are posited to be tight junctions [12, 27, 45, 46], which can be further disintegrated by oxygen deficiency as a result of reduced intestinal perfusion in latent fluid deficiency and LPS-induced systemic inflammation [27, 48, 53], by heat stress [39–41], by intestinal diseases and by high-protein, low-fibre diets [25], whereupon LPS transfer is supposed to increase from mucosal to basolateral transfer [45]. Taking these findings into account, we were able to confirm our third hypothesis: that improving husbandry (e.g., water hygiene and supply and the additional administration of raw fibre) can reduce the clinically assessable SINS score.

Studies on postpartum dysgalactia syndrome in sows (PPDS), a second disease in which bacterial degradation products play a decisive aetiological role [20, 21, 44], demonstrate the outstanding contribution of the coprostatic intestine associated with excessive bacterial colonization and the formation of bacterial degradation products. In addition, the bacterial colonization of the endometrium, the urinary bladder, the teats as well as sepsis and laminitis were identified as causes [22]. The fourth hypothesis was therefore that the clinical quality of the sow, especially the teats, the claws and the occurrence of coprostasis, is associated with SINS in that sow’s offspring. All four hypotheses were tested on offspring from sows with huge quality differences with regards to teats and claws. Tests were conducted in two runs — one with standard husbandry and one with improved water and fibre supply — and in suckling piglets, weaners and fattening pigs.

## Materials and methods

### Experiment and experimental animals

The animal experiment was carried out in the conventional stables of the State Institution for Swine Breeding (Landesanstalt für Schweinezucht, LSZ) Baden-Wurttemburg in Boxberg, Germany under the approval of the authorities in Stuttgart, Baden-Wurttemburg, Germany with file numbers 35–9185.81/0415 and 35–9185.64 / 0035.

A total of 360 pigs were used in the experiment. Nine offspring per sow from 40 sows were successively examined in three age groups: Three piglets per sow as suckling pigs on the third day of life, three others per sow as weanlings 11 days after weaning and the remaining three piglets per sow as fattening pigs during slaughter. All non-slaughtered pigs were euthanized immediately after the clinical examination and sampled for further examinations which were not the subject of this study.

The experiment was carried out in two successive runs (Table 1). The first run was carried out under conventional husbandry conditions (see below). Following the first run, the second run was carried out under improved husbandry conditions (see below). Since the runs were carried out separately, the effects of the targeted changes in husbandry could not be separated from unsystematic housing/feeding effects.

**Table 1:** Experimental setup

	Run 1 (standard husbandry)		Run 2 (improved husbandry)	
Sows’ teats and claws quality	High	Low	High	Low
n sows (litters)	10	10	10	10
n piglets per sow	9	9	9	9
n piglets in total	90	90	90	90
Piglets investigated and taken out d3 p. p.	30 of 90	30 of 90	30 of 90	30 of 90
Piglets investigated and taken out d11 post weaning	30 of 60	30 of 60	30 of 60	30 of 60
Pigs sampled during slaughter	30 of 30	30 of 30	30 of 30	30 of 30

The experiments were performed with offspring of a herd of unique Baden-Wurttemburg Genetics hybrid sows artificially inseminated with Pietrain semen.

### Selection of sows

**Fig. 1:**
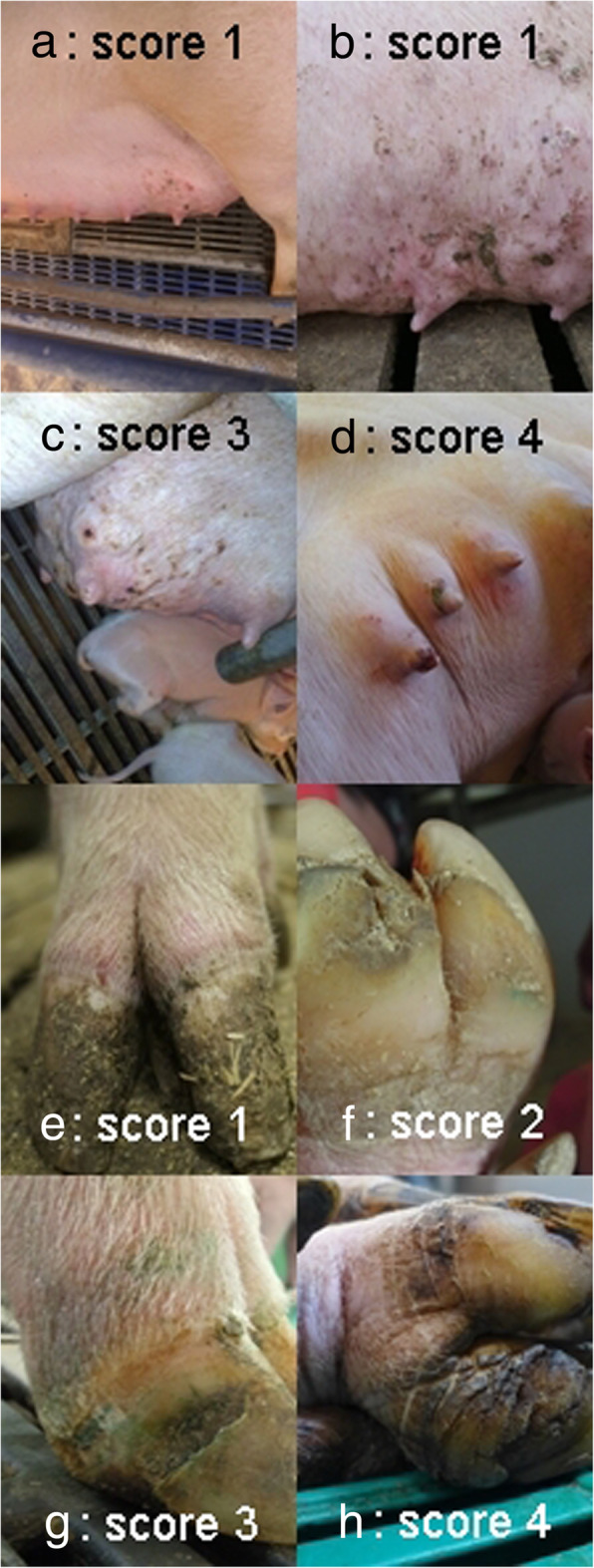
Examples for sows’ teat and claw scores. **a** Slight alterations of the mammary skin (score 1); **b** Efflorations at the mammary skin (score 2); **c** Oedema of the mammary glands (score 3); **d** Vulneration of the teats (score 4); **e** Injury at the coronary band (score 1); **f** Crack at the white line (score 2); **g** Bleeding into the claw wall (score 3); **h** Detachment between sole and heels (score 4)

The sows came from a cohort of 123 animals. Of these, 59 were used for the first run and 64 for the second run. All sows had their external conditions evaluated at the beginning of the experiment (see below). On the 50th day of gestation the condition of the claws and teats were assessed according to [34, 35]. Examples are shown in Fig. 1. For the claws, eight individual characteristics (underdeveloped claws, too long claws, too long dung claws, quality of coronary bands, quality of wall horn, horn cracks, quality of heels and soles, any detachment) were scored from 0 (no clinical deviation) to 4 (strongest deviation) and recorded as mean value per claw. Thus, each animal could achieve values between 0 and 4. Five characteristics for each teat were considered for scoring the mammary glands (alterations to skin and teats; occurrence of rash, oedema or hardening). Each of these findings could have values between 0 and 3. The final score was the sum of all five findings as an average of all existing teats, for possible values between 0 and 15 per sow. The teat score was adjusted to the average of the claw score by a factor of 6 and then multiplied by a factor of 2 due to the higher estimated significance of the teat score versus the claw score. Claw and teat scores were then added to the total sow score.

In the first run, the 10 sows with the lowest total score and 10 sows with the highest total score out of 59 were selected as mothers for the test animals. This procedure was repeated for a second run with 64 sows.

### Housing, feeding and husbandry

#### Standard husbandry conditions

##### Breeding Centre

In the breeding centre, the sows were fixed in stands on a concrete slatted floor until the 28th day of pregnancy. They were fed dry food in the longitudinal trough. Water was available via nipple drinkers.

In the waiting position, the sows stood in a 53 m^2^ compartment on a concrete slatted floor with separate lying areas. Feed could be requested by feeding on demand. Water supply was ensured via nipple and Aqualevel drinking troughs.

The feed for pregnant sows was mixed from barley (69.5%), fibre mix (20%; 27% apple pomace, 25% dried pulp, 25% grass green meal, 20% wheat bran, 3% lignocellulose), high-protein (HP) soybean extraction meal (5%), fish meal (2%), rapeseed oil (1.5%) and a mineral mix (2%; 4% lysine, 0.5% methionine, 0.5% threonine, 19.5% calcium, 3.5% phosphorus). The energy content of the pregnant sows’ feed was calculated as 11.6 MJ metabolizable energy (ME)/kg, with crude protein content of 12.8%.

Three times a year, company’s vaccination husbandry provides stockwise vaccination against *Erysipelothrix rhusiopathiae*, parvovirus (Porcilis Ery + Parvo, MSD) and the EU strain of the porcine reproductive and respiratory syndrome virus (Porcilis PRRS, MSD). Reproductive-oriented vaccination was performed against *Clostridium perfringens* type C and enterotoxic *Escherichia coli* (Clostricol, IDT) on the 85th day of gestation and against *Glaeserella parasuis* (Porcilis Glässer, MSD) on the 100th day of gestation.

##### Farrowing house

In the farrowing house, sows and suckling piglets were kept in 4.8 m^2^ farrowing pens with a honeycomb-shaped plastic-coated metal extended floor. The sows were fixed in a farrowing crate with a flat surface. Feed was offered via volume dosers in the trough. Aqualevel and nipple drinkers ensured the water supply for the animals.

The feed for nursing sows consisted of wheat (45%), barley (24%), HP soybean extraction meal (15%), fibre mix (8%; 27% apple pomace, 25% dried pulp, 25% grass green meal, 20% wheat bran, 3% lignocellulose), fish meal (2%), rapeseed oil (2.5%) and a mineral feed mixture (3.5%; 4% lysine, 0.5% methionine, 0.5% threonine, 19.5% calcium, 3.5% phosphorus). The energy content of the lactation diet was calculated as 13.0 MJ ME/kg, with a crude protein content of 15.9%.

A piglet nest with zone heating and heat lamp was available for the suckling piglets. If a sow’s milk yield was low, milk replacers were provided via troughs. As standard, litters which were too large were adjusted on the farm via a litter compensator with other sows. Suckling pigs that were in the experiment were not transferred. From the second week of life, prestarter was offered to the suckling piglets.

On the day of birth, the tips of the canines of the suckling piglets were ground to minimize injury to each other and the sow. In addition, the piglets received an individual ear tag. The tails were not docked. On the third day of life, the suckling piglets received a subcutaneous iron application to prevent iron deficiency anaemia. As part of vaccination husbandry, piglets were routinely vaccinated against *Mycoplasma hyopneumoniae* (Porcilis M Hyo, MSD) on the third and 21st day of life and against porcine circovirus type 2 (Porcilis PCV, MSD) on the 21st day of life. On the 21st and 28th day of life, the piglets were weighed by default. They were weaned on day 28.

##### Piglet rearing

In piglet rearing, the piglets were placed in 21 m^2^ bays after weaning. The floor structure consisted of plastic and concrete slatted floors in a 50:50 ratio. Feeding and water supply were provided by automatic mixers (Rondomats) and nipple drinkers. In the area of the lying surfaces, the temperature could be adapted to the animals’ heat requirements via a zone heating system with cover (Nürtinger System). The fresh air supply in the compartment was regulated via a porous ceiling with active air extraction. At age approx. Twelve weeks the animals were weighed by default and transferred to the fattening stable.

From the 14th day of life until 10 days after weaning, piglets were fed with “Piglet I”: Barley (34.3%), wheat (23%), oat flakes (10%), rapeseed oil (2%), supplement “FK Hohenlohe” (30%; 30.0% crude protein, 12.0% crude fat, 2.0% crude fibre, 3.6% lysine, 1.6% methionine and cysteine, 2.1% threonine, 0.6% tryptophan, 0.8% valine, 2.0% calcium, 1.1% phosphorus). The energy content of Piglet I was calculated at 13.84 MJ ME/kg, with a crude protein content of 15.9%. “Piglet II” contained barley (29.8%), wheat (39%), soya extraction meal (19.5%), rapeseed oil (2%) and a mineral mix (5%; 10.0% crude protein, 30.0% crude fat, 1.0% lysine, 0.5% methionine + cysteine, 0.6% threonine, 0.1% tryptophan, 0.4% valine, 0.2% calcium, 0.2% phosphorus). Piglet II was administered from day 11 after weaning until the end of the weaning phase. The food was calculated to contain 13.3 MJ ME/kg energy and 17.4% crude protein.

##### Fattening

During the fattening period, the animals were housed in 21 m^2^ and 16 m^2^ bays with concrete slatted floors. Feed and water were accessible via liquid feeding in short troughs and drinking nipples. Ventilation was via a porous ceiling with active air extraction. As soon as the animals reached an estimated weight of approx. 80 kg, they were weighed at regular intervals as standard. They were slaughtered at a bodyweight of approximately 120 kg.

The feeding in the fattening process was two-phase, with a pre-fattening and a final fattening feed. The pre-fattening feed was fed from the second fattening week up to a bodyweight of approx. 80 kg. It contained barley (17%), wheat (59%), soya extraction meal (19%), rapeseed oil (2%) and a mineral feed (3%; 8.0% lysine, 2.0% methionine, 2.0% threonine). The energy content of the pre-fattening feed was calculated at 13.3 MJ ME/kg, with a crude protein content of 17%. Subsequently, the final fattening feed was introduced with barley (62%), wheat (25%), soya extraction meal (10%), rapeseed oil (0.5%) and mineral feed 1 (2.5%). The fattener feed included 12.8 MJ ME/kg energy and 15.3% crude protein.

##### Improved husbandry conditions for the second run

All conditions for feeding and husbandry described thus far apply to the first run. For the second run, the following improvements were made: two bowl drinkers (model 95S-VA, Suevia, Kirchheim /Neckar, Germany) and two conical straw baskets with 38 cm diameter (Hofra, Niederstetten-Adolzhausen) were installed in the waiting stable and straw from LSZ’s own production was passed ad libitum. In the farrowing pen, one mother-child basin drinker (model 20; Suevia, Kirchheim (Neckar)) was installed per farrowing pen. The drinking water was disinfected with chlorine bleaching lye (Ewabo Chemikalien GmbH, Wietmarschen, Germany). Once a day, the sows were given 50 g of straw from LSZ’s own production into the trough.

In the piglet rearing stable, drinking water islands were installed with two bowl drinking troughs per bay (model 95S-VA; Suevia, Kirchheim/Neckar, Germany) and drinking water disinfected with chlorine bleaching lye (Ewabo Chemikalien GmbH, Wietmarschen, Germany). In addition, one hay rack was included per bay (height 60 cm, width 41 cm, length 23.5 cm, slot width 1.8 cm) (Hofra, Niederstetten-Adolzhausen, Germany). Hay from LSZ’s own production was given ad libitum.

In the fattening barn, the bays were each equipped with two bowl drinkers (model 95S-VA, Suevia, Kirchheim/Neckar, Germany) and drinking water disinfected with chlorine bleach (Ewabo Chemikalien GmbH, Wietmarschen, Germany) as well as two 38 cm diameter conical straw baskets (Hofra, Niederstetten-Adolzhausen, Germany). Hay was provided ad libitum from LSZ’s own production.

The water disinfector was connected to the water pipe in every compartment. A TEKNA EVO TPG 603 dosing pump (Seko Deutschland GmbH, Mainz-Kastel, Germany) was used to dose the chlorine bleaching lye. The pump was set to a constant volume of 120 ppm chlorine bleaching lye in the drinking water.

##### Clinical scoring

The sows’ faeces were examined at the time of birth. Any deviations in physiological form and consistency towards clenched, firm faeces were noted as coprostasis (binomial characteristic).

One third of the piglets (30 out of 90) from each litter were randomly selected and clinically evaluated on the third day of life and then euthanized for sampling. Half of the remaining piglets (30 out of 60) were clinically assessed and sampled 11 days after weaning. The remaining piglets (30 of 30) were assessed and sampled at the time of slaughter.

Inflammation and necrosis were clinically assessed as described by Reiner et al. [51]. Due to time constraints and to minimize the animal load, clinical signs were recorded using a digital camera (Lumix, Panasonic Corporation) according to a standardized scheme for later detailed evaluation of the images (Windows Media Player, Version 12, Microsoft GmbH, Germany).

The tail base and tip, ears, teats and navel, coronary bands, wall horn, balls and soles of the feet along with the face were all initially assessed individually. However, the scoring was more detailed than in Reiner et al. [51]. The following clinical characteristics were considered and scored with 0 if the sign was not visible or 1 if the sign was visible. A semiquantitative score was applied if different degrees or values had to be validated. Tail base was scored for length (0 = complete; 1 = 2/3 left; 2 = 1/3 left), swelling (0/1), redness (0/1), rhagades (0/1), exudation (0/1), bleeding (0/1), tail necrosis (0 = none; 1 = partial; 2 = complete tail involved) and ring lacings (0/1). The tail base was separately screened and scored for the presence of bristles (0 = present; 1 = absent), swelling of the tail base (0/1), redness of the tail base (0/1), exudation (0/1) and clinical signs of necrosis (0/1). Ears were scored for the presence of bristles (0 = present; 1 = absent), congested ear veins (0/1) and necrosis of the ears (0 = none; 1 = ear tip, 2 = ear rim). Teats were scored for scab formation (0/1), swelling (0/1), reddening (0/1), necrosis (0/1) and congested blood vessels (0/1). The navel was scored for clinical signs of inflammation (0 = none; 1 = low grade inflammation; 2 = high grade inflammation). The face was scored for oedema around the eyes (0/1) and nasal oedema (0/1). Each claw was individually scored for wall layering (0/1), wall bulging (0/1), wall bleeding (0/1), wall cleavage (0/1), wall cleft (0,1), sole reddening (0/1), detachment of sole from heel (0/1), reddening of heel (0/1), heel cracks (0/1), heel bleeding (0/1), detachment of heel (0/1), redness of coronary band (0/1), exudation of coronary band (0/1) and necrosis of coronary band (0/1). Congestion of the inner thigh veins was also recorded (0/1).

The individual findings were first summarized by organ. Clinical signs of a higher degree were rated more strongly than those of a lower degree (Table 2).

**Table 2:** Addition of single findings to organ scores

Organ	Addition of single findings to organ scores
Claw wall	$\mathsf{CW}\mathsf{Total}=\mathsf{layering}+\left(\mathsf{2}\ \mathrm{x}\ \mathsf{detachment}\right)+\left(\mathsf{3}\ \mathsf{x}\ \mathsf{wall}\ \mathsf{bleeding}\right)+\left(\mathsf{4}\ \mathsf{x}\ \mathsf{wall}\ \mathsf{clevage}\right)+\left(\mathsf{4}\ \mathsf{x}\ \mathsf{wall}\ \mathsf{cleft}\right)$
Coronary band	$\mathsf{CB}\mathsf{Total}=\mathsf{redness}+\left(\mathsf{2}\ \mathrm{x}\ \mathsf{exudation}\right)+\left(\mathsf{3}\ \mathsf{x}\ \mathsf{necrosis}\right)$
Heels	$\mathsf{H}\mathsf{Total}=\mathsf{swelling}+\mathsf{2}\ \mathsf{x}\ \mathsf{redness}+\mathsf{3}\ \mathsf{x}\ \mathsf{cracks}+\mathsf{4}\ \mathsf{x}\ \mathsf{detachment}$
Sole	$\mathsf{So}\mathsf{Total}=\mathsf{redness}+\mathsf{2}\ \mathsf{x}\ \mathsf{detachment}$
Ears	$\mathsf{E}\mathsf{Total}=\mathsf{no}\ \mathsf{bristles}+\mathsf{vein}\ \mathsf{combustion}+\mathsf{necrosis}$
Tail base	$\mathsf{TB}\mathsf{Total}=\mathsf{no}\ \mathsf{bristles}+\mathsf{2}\ \mathsf{x}\ \mathsf{swelling}+\mathsf{3}\ \mathsf{x}\ \mathsf{redness}+\mathsf{4}\ \mathsf{x}\ \mathsf{exudation}+\mathsf{5}\ \mathsf{x}\ \mathsf{necrosis}$
Tail tip	$\mathsf{TT}\mathsf{Total}=\mathsf{no}\ \mathsf{bristles}+\mathsf{2}\ \mathsf{x}\ \mathsf{swelling}+\mathsf{3}\ \mathsf{x}\ \mathsf{redness}+\mathsf{4}\ \mathsf{x}\ \mathsf{rhagades}+\mathsf{5}\ \mathsf{x}\ \mathsf{exudation}+\mathsf{6}\ \mathsf{x}\ \mathsf{bleeding}+\mathsf{7}\ \mathsf{x}\ \mathsf{necrosis}$
Face	$\mathsf{F}\mathsf{Total}=\mathsf{edema}\ \mathsf{around}\ \mathsf{eyes}+\mathsf{nasal}\ \mathsf{edema}$
Teats	$\mathsf{TE}\mathsf{Total}=\mathsf{scab}\ \mathsf{formation}+\mathsf{2}\ \mathsf{x}\ \mathsf{swelling}+\mathsf{3}\ \mathsf{x}\ \mathsf{vein}\ \mathsf{combustion}+\mathsf{4}\ \mathsf{x}\ \mathsf{rhaghades}+\mathsf{5}\ \mathsf{x}\ \mathsf{necrosis}$
Navel	Signs of inflammation (0–2)

The organ scores were combined to give an overall SINS score. The score was calculated separately for each age group. To ensure equal weighting of the organ scores, they were first *z*-transformed within the age group and then added together. The resultant SINS scores corresponded to the normal distribution. The normally distributed values were additionally divided into four quartiles of animals by ascending SINS score.

##### Histological examinations

Ears, claws and tails were removed immediately after death in suckling pigs, weaners and fattening pigs and fixed in 10% formalin. To ensure sufficient fixation of the claws, they were cut open sagittally. An anatomical-pathological examination was performed at the Institute of Veterinary Anatomy (AG Prof. Mülling) and a histopathological examination was performed at the Institute of Veterinary Pathology (AG Prof. Schoon) of Leipzig University.

The claws were processed in accordance with Varagka et al. [64]. The incisions were made in the dorsal area of the wall, halfway between the coronary band and the supporting edge, and perpendicular to the course of the claw bone. An MS100 microtome blade (Micros Produktions- und HandelsgesmbH, St. Veit/Glan, Österreich) was used for the actual sections. The claws of the rearing piglets and fattening pigs were sawn open with an Exakt 300 diamond bandsaw (Exakt Advanced Technologies GmbH, Norderstedt, Germany) with a blade width of 0.2 mm. The claws were cut open so that they could fix better and to ensure the decalcification in Osteomoll® (Merck KGaA, Darmstadt, Germany) was achieved safely.

The ears were sampled at two locations with an MS100 microtome blade (Micros Produktions- und HandelsgesmbH), with at least one sample taken from macroscopically inconspicuous tissue in the area of the ear rim and the other from macroscopically altered locations (if present) or from a further inconspicuous area (if not). Tail samples were taken in the form of a longitudinal section through the distal 1.5 cm from the tip using an MS100 microtome blade (Micros Produktions- und HandelsgesmbH, St. Veit/Glan, Österreich) for suckling pigs and an Exakt 300 diamond bandsaw (Exakt Advanced Technologies GmbH, Norderstedt, Germany) for weaners and fattening pigs, respectively. If lesions were only located proximally further than 1.5 cm from the tip, they were also sampled. Depending on the degree of ossification of the caudal vertebrae, the samples were decalcified with Osteomoll® (Merck KGaA, Darmstadt, Germany) for about 8 h.

##### Paraffin embedding and preparation of histological slides

The histological samples were routinely processed for paraffin embedding. This was performed in an automated procedure using a Hypercenter XP Enclosed Tissue Processor (Thermo Shandon GmbH, Frankfurt am Main, Germany). The samples underwent a defined sequence of chemicals (protocol under 9.1.8). The samples were then transferred into liquid paraffin 56/58 °C (Engelbrecht Medizin und Labortechnik GmbH, Edermünde, Germany) for 240 min and poured into paraffin blocks. Using a Reichert-Jung sled microtome (Optische Werke AG, Vienna, Austria), 3–4 μm thick sections were made and mounted on glass slides (Engelbrecht Medizin und Labortechnik GmbH, Edermünde, Germany). Subsequently, the slides were processed by routine hematoxylin-eosin (HE) staining. The stained specimens were covered with foil using a Tissue-Tek® Film® foil-wrapping machine (Sakura Finetek Germany GmbH, Staufen, Germany). The slides were examined under light microscopy using a BH-2 microscope (Olympus Deutschland GmbH, Hamburg, Germany).

##### Detection of mycotoxins in plasma and bile samples

Blood and bile samples from all euthanized suckling pigs, weaners and fattening pigs were examined for mycotoxins at the Friedrich-Loeffler-Institute for Animal Nutrition, Braunschweig, Germany. Zearalenone (ZEN), DON and their metabolites were analysed in blood plasma in accordance with Brezina et al. [7] and in bile in accordance with Brezina et al. [6] via liquid chromatography-mass spectrometry (LC-MS/MS) using an Agilent 1200 series HPLC system (Agilent Technologies) coupled with a 4000 QTrap LC-MS/MS system (AB Sciex Austria GmbH).

##### Detection of mycotoxins in feed samples

Feed samples were analysed for mycotoxins at the Institute of Bioanalytics and Agro-Metabolomics in the Department of Agrobiotechnology, IFA-Tulln, Austria. The analysis was performed using a multi-mycotoxin method based on HPLC/electrospray ionisation mass spectrometry (HPLC/ESI-MS/MS) [65].

An Agilent 1290 Infinity LC system (Agilent Technologies) was used in combination with a QTrap 5500 LC-MS/MS system (AB Sciex Austria GmbH) to analyse the samples. The analysis included the most important mycotoxins: aflatoxin B1, zearalenone, deoxynivalenol, T-2 toxin, fumonisin B1, ochratoxin A and ergot alkaloids. Additional mycotoxins and metabolites were also determined, including *Alternaria* toxins, *Aspergillus* toxins, bacterial metabolites, depsipeptides, exotic metabolites, *Fusarium* metabolites, *Penicillium* toxins and plant metabolites.

##### Statistics

All statistics were calculated using the Stastistical Package for Social Sciences (SPSS, IBM, Munich, Germany), Version 24. Sows’ scores were normally distributed. The 20 sows with the highest scores and the 20 sows with the lowest at day 50 of gestation were included in the study. Ten each of the sows with highest and lowest scores were studied in each of two consecutive runs, the first under standard husbandry conditions and the second under improved husbandry conditions. When interpreting the husbandry conditions, it is important to note that the specific effects of the fibre and hygienized water cannot be separated from non-specific factors, because the tests under standard conditions had to be performed immediately before the tests under improved conditions. However, no such factors/deviations were identified during the investigations.

For the main part of the experiment, the piglet (weaner, fattening pig) was the statistical unit. All analyses were performed separately for each of the three age groups (suckling piglets, weaners, fatteners). Mean values and standard deviations were calculated with one-way analysis of variance. Prevalence of clinical signs was calculated from binary values (0 = absent; 1 = present). Effects of sow quality (low vs. high) and husbandry (standard vs. improved) on organ scores were calculated using a general linear model. Effects of sow quality (low vs. high) and husbandry (standard vs. improved) on the prevalence of organs involved (0 = not involved; 1 = involved) and on the assessed histological findings (0 = not detected; 1 = detected) were calculated using a binary logistic general linear model. In a second run, this model was extended by the factor coprostasis (0 = normal faecal consistency, 1 = clenched and tight faeces).

Organ scores as shown in Table 2 were *z*-transformed using the “descriptives” routine to adjust the scale of the values of the parameters to be grouped before they were added to the SINS score. *Z*-transformation was performed within each of the three age groups. SINS scores were normally distributed and presented as means together with upper and lower 95%-confidence intervals.

The percentage of explained variance was calculated from the generalized linear models and from a linear regression model including the individual factors and different combinations of the factors. A stepwise linear regression model was used to find the most significant factors for the SINS score in the different age classes. The linear regression models were used to calculate individual and combined effects of husbandry, sow quality and coprostasis on the SINS scores of suckling piglets, weaners and fatteners.

## Results

### Sows

**Fig. 2:**
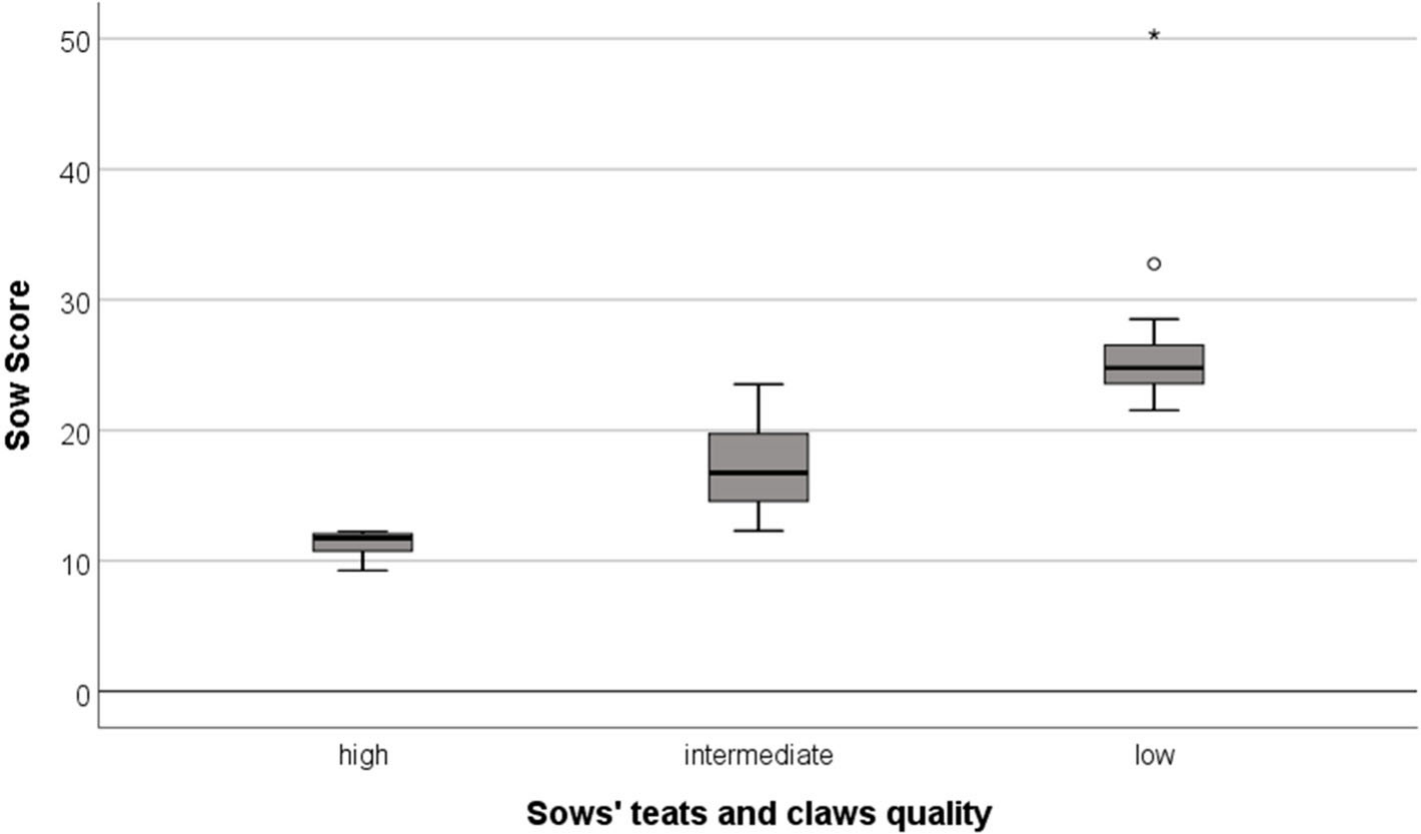
Total scores (quality of teats and claws) of sows with high (*n* = 20) or low (n = 20) quality in teats and claws. Sows with high and low quality were selected as mothers for offspring. Sows with intermediate quality (*n* = 83) were excluded from the study. Star and circle show outliers. Differences in scores between all three groups are statistically significant (*P* < 0.001)

The 20 sows with the lowest and the 20 sows with the highest total scores based on the quality of their teats and claws on the 50th day of gestation were selected as mothers for the experiment. Both groups differed highly significantly in their total score, at 11.3 ± 0.94 vs. 26.4 ± 6.04 (Fig. 2). The sows had a corrected teats score of 1.2 ± 1.0 vs. 13.6 ± 6.1 (factored by 12 to adjust to the claw score) and a claw score of 10.1 ± 0.8 vs. 12.86 ± 1.82. Intermediate sows and their litters were excluded from the study.

All sows with low-quality teats and claws exhibited coprostasis under standard husbandry conditions (100%; *n* = 10), which appeared clinically in the form of solid excrement bales. Among high-quality sows, 70% were affected under standard husbandry conditions. In improved husbandry (water, crude fibre) there was no evidence of coprostasis, regardless of sow quality (0%; data not shown). The combined sow/husbandry effect was statistically significant (*p* < 0.0001). Vaginal discharge was observed in 45% (standard husbandry) and 15% (improved husbandry) of sows, respectively.

### Offspring

Piglets were born weighing 1.69 ± 0.26 kg. They weighed 1.76 ± 0.30 kg 3 days after birth, 7.19 ± 1.22 kg 3 weeks after birth, 10.06 ± 1.61 kg 11 days after weaning and 118.4 ± 6.8 kg at slaughter. There were no significant effects on weight due to sow quality or husbandry conditions. Fifty-one percent of pigs were female and both sexes were equally distributed among groups.

Regardless of the husbandry group, all the suckling piglets showed layers in the claw walls and swelling of the heels. In almost all animals the coronary band was inflammatorily altered, especially with exudation. Inflammatory alterations in teats, ears and in the area of the claws were observed in 56 to 76% of piglets. The base of the tail was affected in 49% of suckling piglets, especially by bristle loss and swelling. Exudation or even necrosis occurred only in exceptional cases. The entire tail was affected in about one third of piglets, mostly by swelling, exudation and scab formation (Table 3).

**Table 3:** Prevalence of clinical signs in different body parts in the age groups studied

Body part	Clinical signs	Suckling piglets	Weaners	Fatteners
Tail Base	no bristles	39.1	19.8	3.9
	swelling	37.4	25.2	2.9
	redness	4.3	2.7	0.0
	exudation	2.6	0.9	1.0
	necrosis	0.9	0.0	0.0
	any	48.7	31.9	4.9
Tail	swelling	18.3	29.7	10.7
	scab formation	14.8	61.3	18.4
	rhagades	9.6	21.6	2.9
	exudation	18.3	58.6	15.5
	necrosis	4.3	36.9	10.7
	ring lacings	0.0	1.8	0.0
	bleeding	1.7	15.3	0.0
	loss	0.0	0.0	45.6
	any	32.2	68.1	21.4
Ears	no bristles	51.3	42.3	6.8
	veins combusted	44.3	69.4	31.1
	necrosis	0.9	2.7	0.0
	any	63.5	76.1	31.1
Face	eye edema	34.8	19.8	0.0
	nasal edema	2.6	3.6	0.0
	any	34.8	19.5	0.0
Teats	veins combusted	48.7	15.3	0.0
	swelling	33.9	27.0	1.0
	redness	8.7	34.2	0.0
	scab formation	41.7	19.8	1.0
	necrosis	4.3	0.9	0.0
	any	75.7	56.6	1.0
Thighs	veins combusted	10.4	0.0	0.0
Navel	inflammation	40.0	4.5	0.0
Coronary bands	redness	58.3	10.8	0.0
	exudation	93.0	8.1	0.0
	nekr	25.2	1.8	0.0
	any	94.8	13.3	0.0
Claw wall	layers	100.0	16.8	0.0
	bulging	34.8	8.8	1.0
	cleavage	0.9	3.5	0.0
	cleft	0.9	5.3	0.0
	bleeding	5.2	32.7	6.8
	any	100.0	41.6	7.8
Soles	redness	55.7	71.7	13.6
	detachment	6.1	28.3	23.3
	any	55.7	74.3	28.2
Heels	swelling	100.0	92.9	61.2
	redness	93.0	61.1	10.7
	cracks	6.1	45.1	82.5
	detachment	0.0	34.5	18.4
	any	100.0	94.7	89.3

**Fig. 3:**
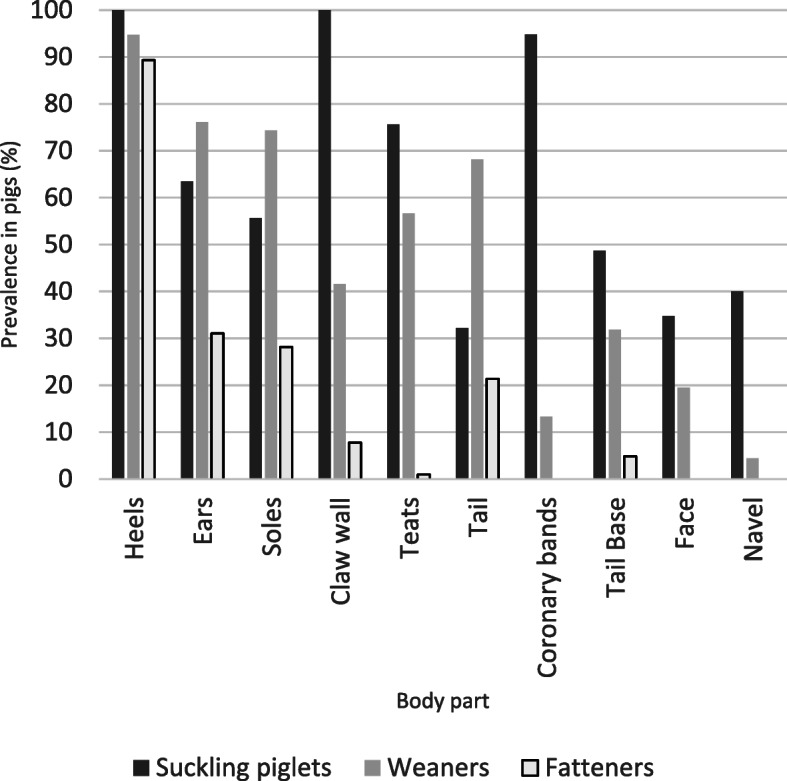
Summary of the clinical signs of inflammation/necrosis in the age groups studied

Weaners were dominated by alterations in heels and signs of inflammation at the ears and soles. Soles and heels were reddened and swollen. Around 60% of the animals showed inflammation in the tail area. Necroses appeared in more than 36% of the animals’ tails. The teats were also affected to a considerable extent. Inflammations had significantly decreased overall (average prevalence 28%) compared to suckling piglets (average prevalence 34.6%). Overall, all parts of the body were frequently affected by aberrations. The suckling piglets and weaners were more uniform than the fattening pigs, where effects on the claws predominated (Fig. 3).

**Fig. 4:**
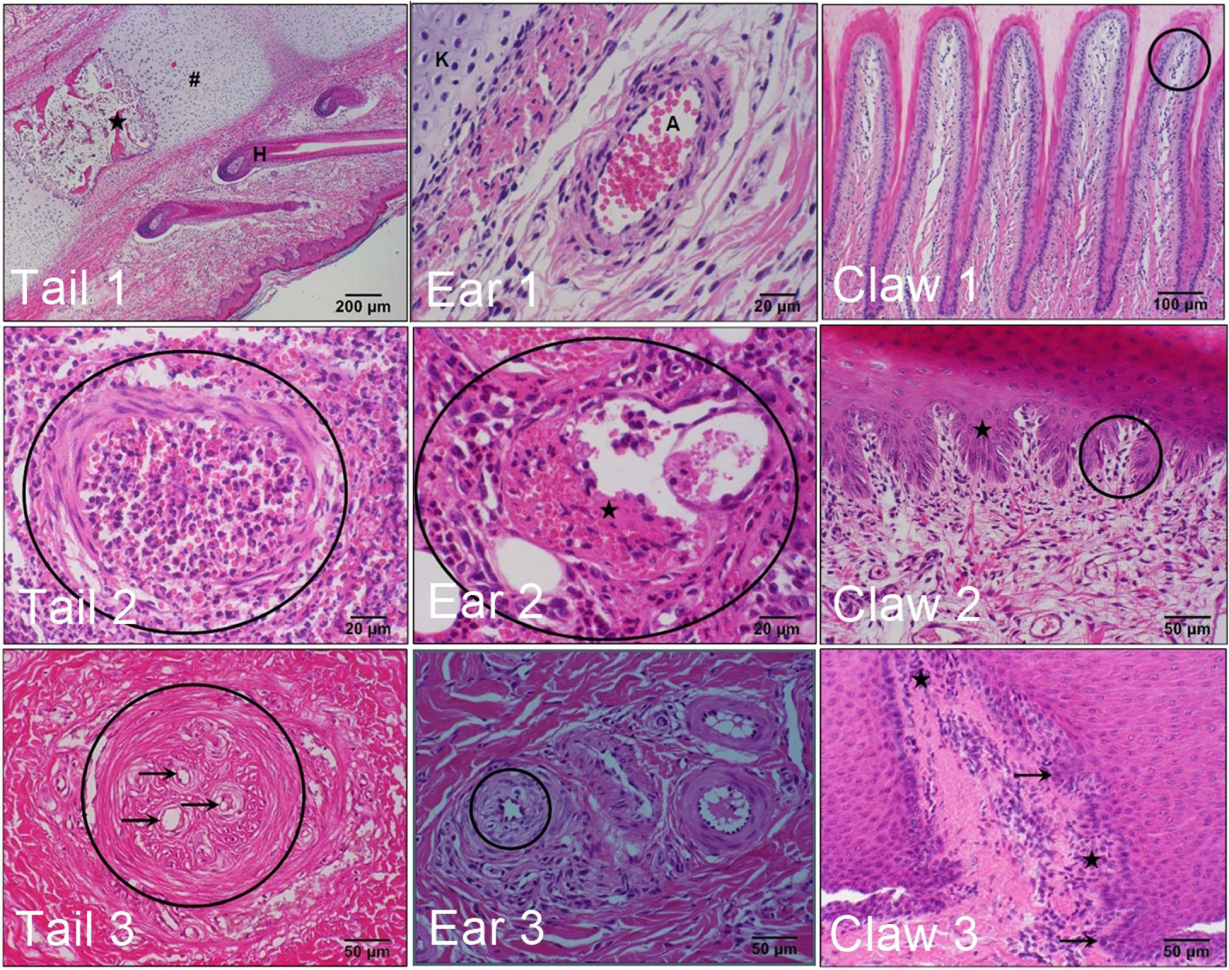
Selected histo-pathological findings in the tail, ear and claw. Tail 1: no specific findings; H: hair follicle, hash: cartilaginous (not yet ossified) part of the caudal vertebrae; asterisk: bony part of the caudal vertebra (with hematopoietically active bone marrow). Tail 2: severe vasculitis (oval). Tail 3: Blood vessel thrombosis; completely organized thrombus (circle) with recanalization (arrows). Ear 1: no specific findings; K: cartilage of the ear, A: arteriole. Ear 2: moderate vasculitis (oval); low-grade vascular thrombosis (asterisk). Ear 3: low-grade subintimal proliferative changes (circle). Claw 1: discreet lymphoplasmacellularinflammation at the tip of dermal laminae (circle). Claw 2: low-grade lymphoplasmacellular inflammation in the dermal laminae (circle); moderate irregular epithelial hyperplasia (asterisk). Claw 3: low grade mixed cell inflammation (predonimatly in the dermis) (asterisks); low grade irregular epithelial hyperplasia (arrows). The shown histological findings could occur in all age groups and with or without alterations of the epithelium (see Tables 4, 5, 6)

Histologically, the tail showed clear signs of inflammation and necrosis in 53, 97 and 100% of the suckling piglets, weaners and fatteners, respectively (Table 4). Epithelial necrosis and purulent inflammation as well as the formation of granulation tissue increased with age. Subepithelial necrosis was most common in weaners. Selected histo-pathological findings in the tail, ear and claw are shown in Fig. 4.

**Table 4:** Histological findings in tails of suckling piglets (SP), weaners (WE) and fatteners (FA)

	SP^a^	WE^b^	FA^c^	SP		WE		FA	
Macroscopically conspicuous	16.52%	92.79%	97.09%	0^d^	1^e^	0^d^	1^e^	0^d^	1^e^
N	115	111	103	93	22	5	106	1	102
Granulation tissue formation: fibroangioblastic	1.74%	16.22%	11.65%	0^f^ (0%)^g^	2 (9.1%)	0 (0%)	18 (17%)	0 (0%)	12 (11.8%)
Granulation tissue formation: mature	0%	15.32%	10.68%	0 (0%)	0 (0%)	0 (0%)	17 (16%)	0 (0%)	11 (10.8%)
Granulation tissue formation: active fibrosis	0%	5.41%	2.91%	0 (0%)	0 (0%)	0 (0%)	6 (5.7%)	1 (100%)	2 (2%)
Granulation tissue formation: inactive fibrosis	0%	4.5%	5.83%	0 (0%)	0 (0%)	0 (0%)	5 (4.7%)	0 (0%)	6 (5.9%)
Inflammation	53.04%	97.3%	100%	39 (41.9%)	22 (100%)	2 (40%)	106 (100%)	1 (100%)	102 (100%)
Inflammation: lymphoplasmacellular	0%	0%	0%	0 (0%)	0 (0%)	0 (0%)	0 (0%)	0 (0%)	0 (0%)
Inflammation: mononuclear	0.87%	0.9%	6.8%	1 (1.1%)	0 (0%)	1 (20%)	0 (0%)	1 (100%)	6 (5.9%)
Inflammation: mixed cellular	1.74%	0%	1.94%	2 (2.2%)	0 (0%)	0 (0%)	0 (0%)	0 (0%)	2 (2%)
Inflammation: purulent	9.57%	63.96%	81.55%	6 (6.5%)	5 (22.7%)	1 (20%)	70 (66%)	0 (0%)	84 (82.4%)
Inflammation: eosinophil	0%	0%	0%	0 (0%)	0 (0%)	0 (0%)	0 (0%)	0 (0%)	0 (0%)
Inflammation: necrotizing	14.78%	1.8%	0.97%	15 (16.1%)	2 (9.1%)	0 (0%)	2 (1.9%)	0 (0%)	1 (1%)
Inflammation: necrosuppurative	23.48%	27.03%	4.85%	14 (15.1%)	13 (59.1%)	0 (0%)	30 (28.3%)	0 (0%)	5 (4.9%)
Necrosis: subepithelial	18.26%	66.67%	36.89%	1 (1.1%)	20 (90.9%)	0 (0%)	74 (69.8%)	0 (0%)	38 (37.3%)
Necrosis: epithelial	19.13%	95.5%	99.03%	0 (0%)	22 (100%)	0 (0%)	106 (100%)	0 (0%)	102 (100%)
Bleeding	33.04%	61.26%	41.75%	25 (26.9%)	13 (59.1%)	0 (0%)	68 (64.2%)	1 (100%)	42 (41.2%)
Bleeding hemosiderosis	0%	0%	0%	0 (0%)	0 (0%)	0 (0%)	0 (0%)	0 (0%)	0 (0%)
Blood vessel thrombosis	15.65%	51.35%	10.68%	16 (17.2%)	2 (9.1%)	0 (0%)	57 (53.8%)	0 (0%)	11 (10.8%)
(Sub) intimal proliferation	56.52%	89.19%	84.47%	53 (57%)	12 (54.5%)	4 (80%)	95 (89.6%)	0 (0%)	87 (85.3%)
Vasculitis	35.65%	40.71%	6.8%	33 (35.5%)	8 (36.4%)	0 (0%)	46 (43.4%)	0 (0%)	7 (6.9%)
Arteriosclerosis	0%	7.21%	71.84%	0 (0%)	0 (0%)	0 (0%)	8 (7.5%)	1 (100%)	73 (71.6%)

^a^, ^b^, ^c^: percentage of suckling piglets (^a^), weaners (^b^) and fattening pigs (^c^) with histological findings. ^d^: individuals without changes of the epithelium; ^e^: individuals with changes to the epithelium. ^f^: absolute number of individuals with histological findings; ^g^: percentage of individuals with histological findings (in brackets)

Tissue bleeding occurred in suckling pigs and fattening pigs in a similar manner. Vasculitis was present in all age groups, but especially in suckling piglets and weaners. Thrombolization of the blood vessels also occurred regularly. Arteriosclerotic-like lesions were only observed in weaners and fattening pigs, with the latter group particularly affected. The right-hand side of the table compares the findings of cuts without (0) and with (1) alterations to the epithelium. In weaners and fattening pigs, only individual animals without alterations were found. In suckling piglets, however, the epithelium was not affected in 93/115 animals. Nevertheless, 57% of the piglets showed intima proliferation, 17% thrombosis and 35.5% vasculitis. Necrosis occurred in 16% of the animals. In contrast, areas of purulent inflammation were less frequent than in animals with defects in the epidermis. Similar findings, but to a much lesser extent than on the tail, were found for the ears (Table 5). In addition, in the claw area a strong increase was observed in external damage from suckling piglets via weaners to fattening pigs (Table 6). However, inflammation and necrosis occurred most frequently in suckling pigs and weaners. Parakeratotic changes were also observed in the suckling piglets in particular.

**Table 5:** Histological findings in ears of suckling piglets (SP), weaners (WE) and fatteners (FA)

	SP^a^	WE^b^	FA^c^	SP		WE		FA	
Macroscopically conspicuous	14.78	76.58	63.11	0^d^	1^e^	0^d^	1^e^	0^d^	1^e^
n	115	111	103	98	17	29	82	44	59
Granulation tissue formation: fibroangioblastic	13.91	76.58	63.11	1 (1)	16^f^ (94.1%^g^)	5 (17.2%)	80 (97.6%)	7 (15.9%)	58 (98.3%)
Granulation tissue formation: mature	0	0	0	0 (0)	16 (94.1%)	5 (17.2%)	80 (97.6%)	7 (15.9%)	58 (98.3%)
Granulation tissue formation: active fibrosis	0	0	0	0 (0)	0 (0%)	0 (0%)	0 (0%)	0 (0%)	0 (0%)
Granulation tissue formation: inactive fibrosis	0	0	0	0 (0)	0 (0%)	0 (0%)	0 (0%)	0 (0%)	0 (0%)
Inflammation	5.22	56.76	34.95	0 (0)	0 (0%)	0 (0%)	0 (0%)	0 (0%)	0 (0%)
Inflammation: lymphoplasmacellular	0	0	0	1 (1)	5 (29.4%)	2 (6.9%)	61 (74.4%)	3 (6.8%)	33 (55.9%)
Inflammation: mononuclear	0	0	0	0 (0)	0 (0%)	0 (0%)	0 (0%)	0 (0%)	0 (0%)
Inflammation: mixed cellular	0.87	7.21	6.8	0 (0)	0 (0%)	0 (0%)	0 (0%)	0 (0%)	0 (0%)
Inflammation: purulent	14.78	72.07	61.17	0 (0)	1 (5.9%)	4 (13.8%)	4 (4.9%)	3 (6.8%)	4 (6.8%)
Inflammation: eosinophil	0	0	0	1 (1)	16 (94.1%)	3 (10.3%)	77 (93.9%)	8 (18.2%)	55 (93.2%)
Inflammation: necrotizing	0	0	0	0 (0)	0 (0%)	0 (0%)	0 (0%)	0 (0%)	0 (0%)
Inflammation: necrosuppurative	0	0	0	0 (0)	0 (0%)	0 (0%)	0 (0%)	0 (0%)	0 (0%)
Necroses: subepithelial	11.3	34.23	39.81	0 (0)	0 (0%)	0 (0%)	0 (0%)	0 (0%)	0 (0%)
Necrosis: epithelial	14.78	73.87	57.28	0 (0)	13 (76.5%)	0 (0%)	37 (45.1%)	0 (0%)	41 (69.5%)
Bleeding	1.74	18.92	8.74	0 (0)	17 (100%)	0 (0%)	82 (100%)	0 (0%)	59 (100%)
Bleeding hemosiderosis	0	0	0	0 (0)	2 (11.8%)	2 (6.9%)	19 (23.2%)	0 (0%)	9 (15.3%)
Blood vessel thrombosis	0.87	4.5	1.94	0 (0)	0 (0%)	0 (0%)	0 (0%)	0 (0%)	0 (0%)
(Sub) intimal proliferation	0	2.7	1.94	0 (0)	1 (5.9%)	0 (0%)	5 (6.1%)	0 (0%)	2 (3.4%)
Vasculitis	3.48	27.93	33.98	0 (0)	0 (0%)	0 (0%)	3 (3.7%)	0 (0%)	2 (3.4%)
Arteriosclerosis	0	0	0	0 (0)	4 (23.5%)	0 (0%)	31 (37.8%)	1 (2.3%)	34 (57.6%)

^a^, ^b^, ^c^: percentage of suckling piglets (^a^), weaners (^b^) and fattening pigs (^c^) with histological findings. ^d^: individuals without changes of the epithelium; ^e^: individuals with changes to the epithelium. ^f^: absolute number of individuals with histological findings; ^g^: percentage of individuals with histological findings (in brackets)

**Table 6:** Histological findings in claws of suckling piglets (SP), weaners (WE) and fatteners (FA)

	SP^a^	WE^b^	FA^c^	SP		WE		FA	
Macroscopic conspicuous	17.39%	44.14%	95.15%	0^d^	1^e^	0^d^	1^e^	0^d^	1^e^
n	115	111	103	95	20	62	49	5	98
Inflammation	21.74%	22.52%	5.83%	7^f^ (7.4%^g^)	18 (90%)	10 (16.1%)	15 (30.6%)	3 (60%)	3 (3.1%)
Inflammation: lymphoplasmacellular	6.09%	15.32%	4.85%	7 (7.4%)	0 (0%)	10 (16.1%)	7 (14.3%)	3 (60%)	2 (2%)
Inflammation: mononuclear	0%	0%	0%	0 (0%)	0 (0%)	0 (0%)	0 (0%)	0 (0%)	0 (0%)
Inflammation: mixed cellular	2.61%	1.8%	0%	1 (1.1%)	2 (10%)	0 (0%)	2 (4.1%)	0 (0%)	0 (0%)
Inflammation: purulent	13.91%	3.6%	0%	1 (1.1%)	15 (75%)	0 (0%)	4 (8.2%)	0 (0%)	0 (0%)
Inflammation: eosinophil	0%	0%	0%	0 (0%)	0 (0%)	0 (0%)	0 (0%)	0 (0%)	0 (0%)
Inflammation: necrotizing	0%	0%	0%	0 (0%)	0 (0%)	0 (0%)	0 (0%)	0 (0%)	0 (0%)
Inflammation: necrosuppurative	0.87%	0.9%	0%	0 (0%)	1 (5%)	0 (0%)	1 (2%)	0 (0%)	0 (0%)
Necrosis	15.65%	5.41%	0.97%	1 (1.1%)	17 (85%)	0 (0%)	6 (12.2%)	0 (0%)	1 (1%)
Spatial association between inflammation and necrosis	15.65%	5.41%	0.97%	1 (1.1%)	17 (85%)	0 (0%)	6 (12.2%)	0 (0%)	1 (1%)
Bleeding	20%	2.7%	0%	13 (13.7%)	10 (50%)	3 (4.8%)	0 (0%)	0 (0%)	0 (0%)
Bleeding hemosiderosis	0%	0%	0%	0 (0%)	0 (0%)	0 (0%)	0 (0%)	0 (0%)	0 (0%)
Epithelial hyperplasia	66.09%	68.47%	9.71%	58 (61.1%)	18 (90%)	49 (79%)	27 (55.1%)	5 (100%)	5 (5.1%)
Epithelial hyperplasia: regular	0.87%	0%	0%	0 (0%)	1 (5%)	0 (0%)	0 (0%)	0 (0%)	0 (0%)
Epithelial hyperplasia: irregular	65.22%	68.47%	10.68%	58 (61.1%)	17 (85%)	49 (79%)	27 (55.1%)	5 (100%)	6 (6.1%)
Epithelial hyperplasia: papillary	0%	0%	0%	0 (0%)	0 (0%)	0 (0%)	0 (0%)	0 (0%)	0 (0%)
Epithelial hyperplasia: psoriasiform	0%	0.9%	0%	0 (0%)	0 (0%)	0 (0%)	1 (2%)	0 (0%)	0 (0%)
Epithelial hyperplasia: pseudocarcinomatous	0%	0%	0%	0 (0%)	0 (0%)	0 (0%)	0 (0%)	0 (0%)	0 (0%)
Epithelial hyperplasia papillary irregular	0%	0%	0%	0 (0%)	0 (0%)	0 (0%)	0 (0%)	0 (0%)	0 (0%)
Epithelial hyperplasia papillary regular	0%	0%	0%	0 (0%)	0 (0%)	0 (0%)	0 (0%)	0 (0%)	0 (0%)
Hyperkeratosis	13.91%	5.41%	0.97%	1 (1.1%)	15 (75%)	1 (1.6%)	5 (10.2%)	0 (0%)	1 (1%)
Hyperkeratosis: orthokeratotic	0%	1.8%	0.97%	0 (0%)	0 (0%)	1 (1.6%)	1 (2%)	0 (0%)	1 (1%)
Hyperkeratosis: parakeratotic	13.91%	3.6%	0%	1 (1.1%)	15 (75%)	0 (0%)	4 (8.2%)	0 (0%)	0 (0%)
Claw papilliform structure	0%	0%	0%	0 (0%)	0 (0%)	0 (0%)	0 (0%)	0 (0%)	0 (0%)

^a^, ^b^, ^c^: percentage of suckling piglets (^a^), weaners (^b^) and fattening pigs (^c^) with histological findings. ^d^: individuals without changes of the epithelium; ^e^: individuals with changes to the epithelium. ^f^: absolute number of individuals with histological findings; ^g^: percentage of individuals with histological findings (in brackets)

### Effects of sow quality and husbandry

For the individual parameters (Tables 7, 8, 9), significant influences of husbandry and sow quality were seen. This was particularly notable for the scores, but was also seen in the proportion of affected animals. Both effects were most evident in weaners, but also in suckling piglets. The effects of husbandry could be followed until the fattening phase. There was already a trend that sow quality was much less decisive with improved husbandry than with standard husbandry. Almost every part of the body was affected in suckling piglets. This was even clearer in weaners.

**Table 7:** Effects of sows’ quality and husbandry on organ scores and prevalences of clinical signs of inflammation and necrosis in suckling piglets

Husbandry	Standard			Improved			
Sows’ quality	Low	High	P_sow_	Low	High	P_sow_	P_total_
Organ scores							
Tail Base	2.04 ± 0.39	2.07 ± 0.39	n.s.	0.87 ± 0.36	0.74 ± 0.39	n.s.	0.01
Tail tip	3.18 ± 0.61	3.04 ± 0.61	n.s.	0.56 ± 0.57	0.74 ± 0.62	n.s.	0.001
Ears	1.46 ± 0.14	1.25 ± 0.14	n.s.	0.69 ± 0.13	0.48 ± 0.14	n.s.	< 0.0001
Claw wall	14.71 ± 0.39	14.68 ± 0.39	n.s.	12.56 ± 0.37	12 ± 0.39	n.s.	< 0.0001
Coronary bands	10.79 ± 1.17	12.64 ± 1.17	n.s.	6.69 ± 1.09	9.89 ± 1.19	0.04	0.002
Soles	5.5 ± 0.40	2.93 ± 0.42	< 0.0001	0.94 ± 0.37	0.48 ± 0.42	< 0.0001	< 0.0001
Heels	18.46 ± 1.02	19.25 ± 1.02	n.s.	21 ± 0.96	19.07 ± 1.04	n.s.	n.s.
Teats	5.11 ± 0.62	3.71 ± 0.62	n.s.	4.31 ± 0.58	2.63 ± 0.63	0.04	0.04
Prevalences							
Tail Base	68 ± 8.8	64 ± 9.1	n.s.	38 ± 8.6	26 ± 8.4	n.s.	0.001
Tail tip	57 ± 9.4	46 ± 9.4	n.s.	9 ± 5.2	19 ± 7.5	n.s.	< 0.0001
Ears	93 ± 4.9	79 ± 7.8	n.s.	50 ± 6.8	33 ± 10.1	n.s.	< 0.0001
Claw wall	100 ± 0	100 ± 0	n.s.	100 ± 0	100 ± 0	n.s.	n.s.
Coronary bands	96 ± 3.5	100 ± 0	n.s.	88 ± 5.8	96 ± 3.6	n.s.	n.s.
Soles	100 ± 0	54 ± 9.4	< 0.0001	41 ± 8.7	30 ± 8.8	n.s.	< 0.0001
Heels	100 ± 0	100 ± 0	n.s.	100 ± 0	100 ± 0	n.s.	n.s.
Teats	89 ± 5.8	71 ± 8.5	n.s.	78 ± 7.3	63 ± 9.3	n.s.	n.s.

**Table 8:** Effects of sows’ quality and husbandry on organ scores and prevalences of clinical signs of inflammation and necrosis in weaners

Husbandry	Standard			Improved			
Sows’ quality	Low	High	Psow	Low	High	Psow	Ptotal
Organ scores							
Tail Base	2.07 ± 0.20	1.18 ± 0.20	n.s.	0 ± 0.19	0 ± 0.23	n.s.	< 0.0001
Tail tip	11.93 ± 1.13	8.04 ± 1.13	n.s.	3.53 ± 1.06	6.96 ± 1.25	n.s.	< 0.0001
Ears	1.5 ± 0.14	1.18 ± 0.14	n.s.	0.88 ± 0.13	1.04 ± 0.16	n.s.	0.01
Claw wall	9.57 ± 1.19	6.18 ± 1.19	0.04	0.75 ± 1.11	1.04 ± 1.31	n.s.	< 0.0001
Coronary bands	1.43 ± 0.27	0.4 3 ± 0.27	0.009	0.25 ± 0.25	0 ± 0.30	n.s.	0.002
Soles	5.57 ± 0.69	3.21 ± 0.69	0.02	2.53 ± 0.65	3.22 ± 0.76	n.s.	0.009
Heels	24.14 ± 2.73	20.21 ± 2.73	n.s.	16 ± 2.56	13.7 ± 3.01	n.s.	0.04
Teats	4.36 ± 0.51	2.54 ± 0.51	0.01	2.31 ± 0.48	0.78 ± 0.56	0.05	< 0.0001
Prevalences							
Tail Base	75 ± 8.2	54 ± 9.4	n.s.	0 ± 0	0 ± 0	n.s.	< 0.0001
Tail tip	96 ± 3.5	82 ± 7.2	n.s.	47 ± 8.8	52 ± 10.4	n.s.	< 0.0001
Ears	96 ± 3.5	79 ± 7.8	n.s.	63 ± 8.6	74 ± 9.2	n.s.	< 0.0001
Claw wall	68 ± 8.8	68 ± 8.8	n.s.	16 ± 6.4	17 ± 7.9	n.s.	< 0.0001
Coronary bands	32 ± 8.8	18 ± 7.2	n.s.	3 ± 3.1	0 ± 0	n.s.	< 0.0001
Soles	86 ± 6.6	75 ± 8.2	n.s.	69 ± 8.2	74 ± 9.2	n.s.	n.s.
Heels	100 ± 0	100 ± 0	n.s.	91 ± 5.2	96 ± 4.3	n.s.	n.s.
Teats	79 ± 7.8	71 ± 8.5	n.s.	47 ± 8.8	30 ± 9.6	n.s.	< 0.0001

**Table 9:** Effects of sows’ quality and husbandry on organ scores and prevalences of clinical signs of inflammation and necrosis in fatteners

Husbandry	Standard			Improved			
Sows’ quality	Low	High	Psow	Low	High	Psow	Ptotal
Organ scores							
Tail Base	0.28 ± 0.14	0.29 ± 0.15	n.s.	0 ± 0.13	0 ± 0.15	n.s.	n.s.
Tail tip	4.08 ± 0.73	3.58 ± 0.75	n.s.	0 ± 0.67	0 ± 0.75	n.s.	< 0.0001
Ears	0.36 ± 0.12	0.58 ± 0.12	n.s.	0.2 ± 0.11	0.42 ± 0.12	n.s.	n.s.
Claw wall	0.44 ± 0.18	0.63 ± 0.18	n.s.	0 ± 0.16	0 ± 0.18	n.s.	0.02
Coronary bands	0 ± 0	0 ± 0	n.s.	0 ± 0	0 ± 0	n.s.	n.s.
Soles	1.92 ± 0.41	2 ± 0.42	n.s.	0.47 ± 0.37	0.17 ± 0.42	n.s.	0.001
Heels	23.36 ± 2.63	18.17 ± 2.68	n.s.	19.63 ± 2.39	12.92 ± 2.68	n.s.	0.05
Teats	0 ± 0.06	0.13 ± 0.06	n.s.	0 ± 0.05	0 ± 0.06	n.s.	n.s.
Prevalences							
Tail Base	8 ± 5.4	12 ± 6.8	n.s.	0 ± 0	0 ± 0	n.s.	n.s.
Tail tip	52 ± 10	38 ± 9.9	n.s.	0 ± 0	0 ± 0	n.s.	< 0.0001
Ears	36 ± 9.6	33 ± 9.6	n.s.	17 ± 6.8	42 ± 10.1	n.s.	n.s.
Claw wall	16 ± 7.3	17 ± 7.6	n.s.	0 ± 0	0 ± 0	n.s.	0.02
Coronary bands	0 ± 0	0 ± 0	n.s.	0 ± 0	0 ± 0	n.s.	n.s.
Soles	48 ± 10	50 ± 10.2	n.s.	10 ± 5.5	8 ± 5.6	n.s.	< 0.0001
Heels	96 ± 3.9	92 ± 5.6	n.s.	83 ± 6.8	88 ± 6.8	n.s.	n.s.
Teats	0 ± 0	4 ± 4.1	n.s.	0 ± 0	0 ± 0	n.s.	n.s.

**Fig. 5:**
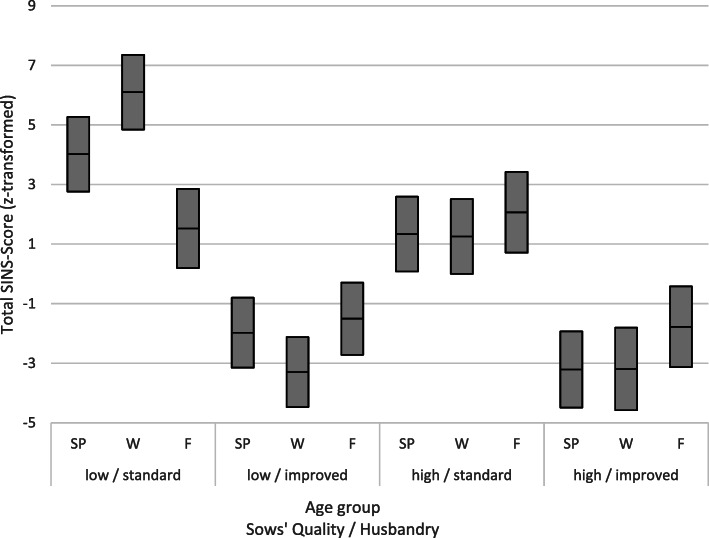
The total SINS-score based on z-transformed organ scores as a function of age group (SP = suckling piglets; W = weaners; F = fatteners), low or high quality of the sow (quality of their teats and claws) and the quality of the husbandry (standard vs. improved). Each Box shows the Mean and the upper and lower 95% confidence interval. SINS-scores of the low/standard group are significantly different from all other groups in any age class. Scores of the low/improved and the high/improved groups are never significantly different in none of the age classes. Scores of the high/standard group differ significantly from those of any other groups in suckling piglets and weaners, but not from the low/standard group in fatteners

To summarize the effects of the individual parameters in the sense of SINS, the findings for the individual organ systems were summarized according to Table 2, then *z*-transformed in order to adjust the different variables before being added to the SINS score. Figure 5 shows both the mean values and the upper and lower limits of the 95%-confidence intervals of the SINS scores as a function of age group, sow quality and quality of husbandry. By far the highest SINS scores occurred in the offspring of sows with poor quality teats and claws under standard husbandry conditions (no extra water, no additional fibre). Weaners were most affected, closely followed by suckling piglets. The lowest SINS grades were found in fattening pigs. With improved husbandry, suckling and weaned piglets saw a significant decline in SINS scores, but this was not observed for fatteners. The decline in SINS scores was even more pronounced and statistically highly significant in all age groups when using top-quality mothers, as judged by the health of their teats and claws. The strongest improvement was achieved when both sow quality and husbandry were improved.

The offspring of low-quality sows exhibited a difference in standard and improved husbandry conditions of 6.0, 9.4 and 3.0 points for suckling piglets, weaners and fattening pigs, respectively. In the offspring of high-quality sows, these differences shrank to 4.5, 4.0 and 3.8 points for suckling piglets, weaners and fattening pigs, respectively. Conversely, the improvement in sow quality under standard husbandry conditions led to a decrease in SINS score of 2.7, 5.3 and − 0.5 points in suckling pigs, weaners and fattening pigs, respectively. The lowest effects were achieved by improving the sow quality under improved husbandry conditions: only 1.2, − 0.1 and 0.3 points in suckling piglets, weanlings and fattening pigs, respectively. Table 10 shows these associations based on the percentage effects, where the highest effect of improving husbandry conditions in weaners from low-quality sows was set to 100%. It shows that effects in suckling piglets and fatteners, respectively, reached on average 77 and 35% of those of the weaners. Across all age groups, the improvement in husbandry conditions for low-quality sows accounted for 65% of the maximum observed reduction in the SINS score, while this figure was 44% for high-quality sows. In contrast, the improvement in sow quality under standard husbandry conditions only resulted in a 26% reduction in the SINS score, with a 5% reduction under improved husbandry conditions.

**Table 10:** Percentage effects of improving sow quality or husbandry conditions in front of different sow/husbandry-backgrounds. All data related to the maximum effect (100%) achieved in weaners by improvement of husbandry conditions in low quality sows

Step	Improving housing	Improving housing	Improving sow q	Improving sow q	Improving both	
in	Low Q sow	High Q sow	Standard housing	Improved housing	LowQ +standard housing	Mean per age class
SP	63.8	48.4	28.5	13.2	76.9	46.2
W	100.0	42.9	56.0	−1.1	98.9	59.3
F	32.2	40.9	−5.8	2.9	35.1	21.1
Mean all age classes	65.3	44.1	26.3	5.0	70.3	

*SP* Suckling piglets, *W* Weaners, *F* Fatteners, *Q* Quality

In the suckling piglets without SINS signs (data not shown) only 10.3% of the sows showed signs of coprostasis. In contrast, for the offspring with the strongest SINS signs, 77.8% of the sows were affected by coprostasis. This relationship was statistically highly significant (*p* < 0.001). In weaners, the association was even more extreme, at 4.3 and 89.3% (*p* < 0.001). Even in the fattening pigs, which had no animals among the lowest SINS scores, the association with 32.4% (group of offspring with moderate SINS signs) and 64.3% (group of offspring with highest degree in SINS) of sows with coprostasis was still significant (*p* = 0.033).

**Fig. 6:**
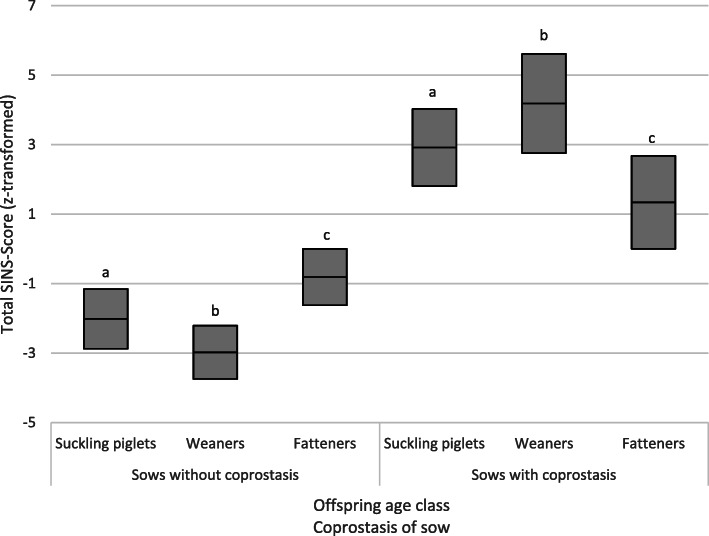
Effects of sow coprostasis on SINS scores in suckling piglets, weaners and fatteners. The boxes show the mean value and the upper and lower values of the 95% confidence interval. Groups with the same letter above the boxes differed significantly with P < = 0.007

The *z*-transformed SINS scores of suckling piglets, weaners and fatteners from sows without coprostasis were on average − 2.0, − 3.0 and − 0.8, respectively (Fig. 6). In offspring of sows with coprostasis, the SINS values were significantly higher (*p* < 0.001 in suckling piglets and weaners; *p* = 0.007 in fatteners). The mean values for suckling piglets, weaners and fatteners were now 2.9, 4.2 and 1.2 points, respectively. The coprostasis explained 31, 45 and 7% of the variance in the SINS score in suckling piglets, weaners and fatteners, respectively.

**Fig. 7:**
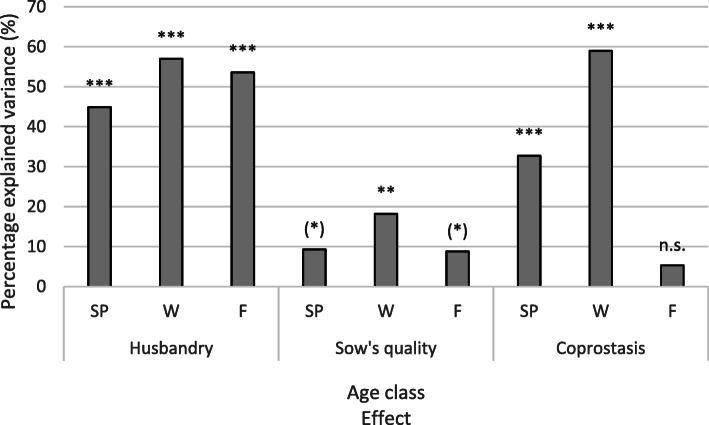
Proportion of explained variance for the average z-transformed SINS score of the sows’ offspring by husbandry (standard vs. improved), quality of sow (teats and claws; low vs. high quality) and findings of coprostasis in the sow (with vs. without coprostasis). The results are considered separately by age group (SP: suckling piglets; W: weaners; F; fatteners). Significance of the effect: (*): *P* = 0.67; **: *P* < 0.01; ***: *P* < 0.001; n.s.: not significant

The effect of husbandry was significant in all three age groups with a correspondingly high explained variance (Fig. 7). This characteristic explained between 45 and 57% of the variance in the *z*-transformed SINS scores of the offspring. The effects of sow teat and claw quality were much lower, at 9 to 18% explained variance. The presence or absence of coprostasis in sows during early puerperium explained 33% of the SINS variance for the suckling piglets and 59% of the variance for the weaners, but showed no effects on the SINS scores of the fattening pigs. The effects on the weaners were most pronounced in all areas.

In the stepwise linear regression with the SINS score as the dependent variable, the effects for husbandry and sow quality were significant in the suckling piglets (Table 11). Together they explained 57% of the variance in the SINS score. Three effects were considered in the weaners: coprostasis, sow quality and husbandry, with an explained total variance of 68%. For fattening pigs, 58% of the variance in the SINS score was explained by the effect of husbandry.

**Table 11:** Results of a stepwise linear regression model on the dependent variable SINS-score, including husbandry, sow’s quality and coprostasis as independent variables

Age Class	Independent variables	R	R-Square	P
Suckling	Husbandry	0.681	0.464	< 0.001
piglets	Sow’s quality	0.756	0.572	< 0.001
Weaners	Coprostasis	0.764	0.583	< 0.001
	Sow’s quality	0.801	0.642	< 0.001
	Husbandry	0.827	0.684	< 0.001
Fatteners	Husbandry	0.580	0.336	< 0.001

**Fig. 8:**
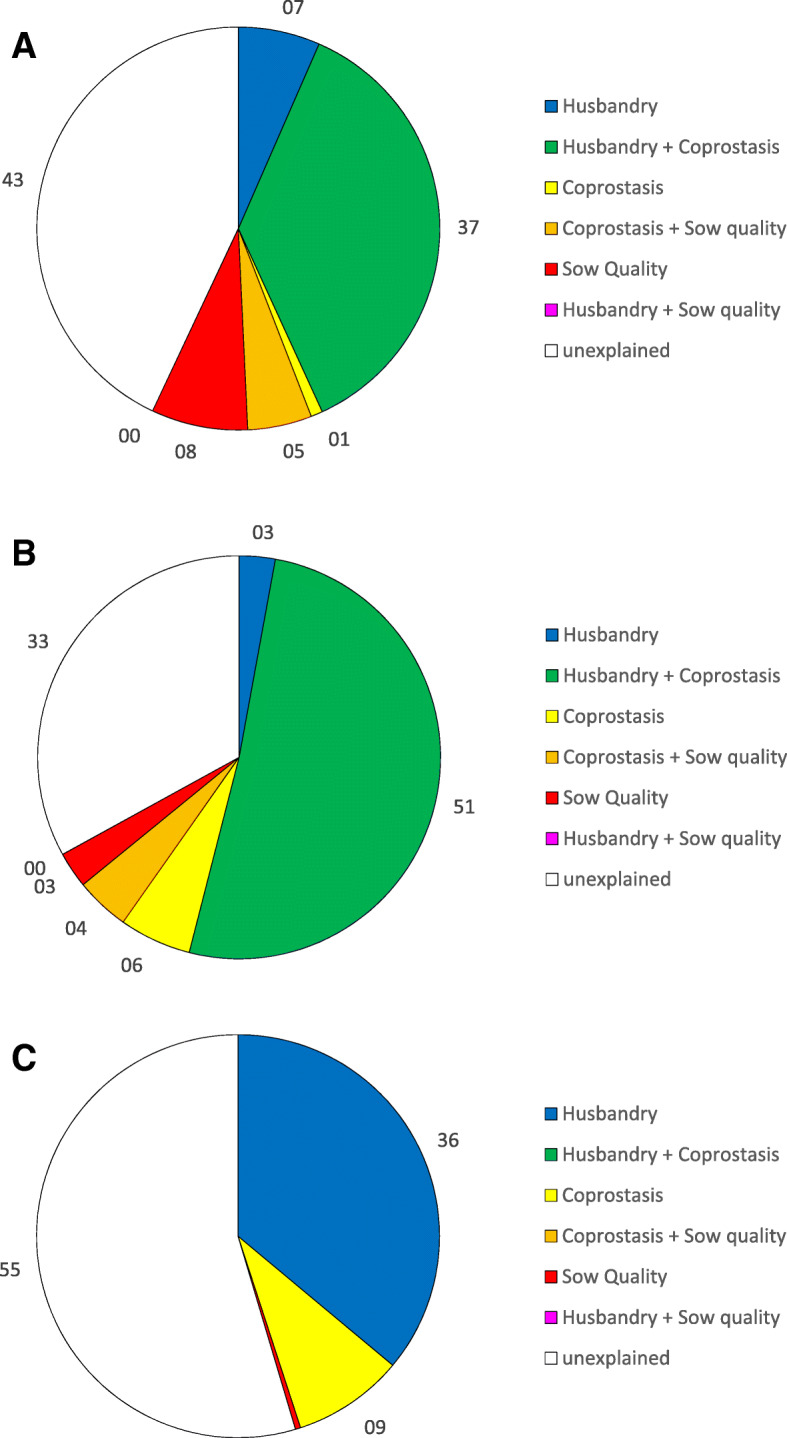
Variance of the SINS score explained by single and combined effects of husbandry, sows’ quality and coprostasis in suckling piglets

Figure 8 shows the shares of SINS variance explained by the individual effects of husbandry, coprostasis and sow quality and by combined/overlapping effects (husbandry/coprostasis and sow quality/coprostasis). There was no combined effect between husbandry and sow quality, as both effects were absolutely independent from each other (0% common variance). For example, in weaners, 33% of variance was unexplained. More than 50% of variance was explained by the overlapping of husbandry and coprostasis (both factors overlapped by 74%). Thus, 54% of SINS variance was explained by the husbandry, 51.5% by the combined effect with coprostasis and 2.9% by an effect independent from coprostasis. Coprostasis explained 51.1 +  5.8 +  4.3 = 61.2% of SINS variance, alone or in combination with husbandry and sow quality in weaners.

The analysis of the feed samples (*n* = 15) showed the presence of a large variety of mycotoxins, of which only some will be further discussed. The most researched and frequently occurring mycotoxins are ZEN, DON, T-2 toxin, aflatoxins, fumonisins, ochratoxin A and ergot alkaloids. While aflatoxins, fumonisins and ochratoxin A were not detected in any of the analysed feed samples, T-2 toxin (up to 144.8 μg/kg) and total ergot alkaloids (EA) (up to 174.1 μg/kg) were present in more than half of the analysed samples. The *Fusarium* toxins ZEN (MIN – MAX; 3.0–45.0 μg/kg) and DON (57.5–302.9 μg/kg) showed positive test results in all samples. Furthermore, various other *Fusarium* toxins such as enniatin (up to 703.5 μg/kg), aurofusarin (up to 616.9 μg/kg) or culmorin (up to 405.5 μg/kg) were present in all feed samples. Other mycotoxins from mycotoxigenic fungal genera like *Alternaria* or *Aspergillus* were also frequently detected.

ZEN was found in almost all samples of bile and plasma, with the ZEN concentration in bile about 5 times higher compared to in plasma. The concentration ranged between < 0.02 ng/mL and 0.1 ng/mL in plasma and < 0.01 ng/mL and 3.27 ng/mL in bile. Alpha-zearalenol (α-ZEL) was detected in two bile samples with a concentration up to 0.34 ng/mL. Further metabolites of ZEN as well as DON (including de-epoxy-DON) were lower than the limit of detection.

## Discussion

Inflammation and necrosis are important causes of pain, suffering and harm to animals. Avoiding these negative consequences is one of the great challenges of any future-oriented animal husbandry. Swine inflammation and necrosis syndrome (SINS) is a newly identified clinical syndrome in which the transfer of bacterial products (e.g., LPS) into the bloodstream is hypothesized to be responsible for the development of inflammation and necrosis in the extremities [50, 51]. Body parts such as tail, ears, teats, navel, coronary bands, claw wall, sole and heels seem to be affected regularly. Some of these signs can be explained by aggressive biting or technopathies, e.g., due to unfavourable floor, but they have also frequently been identified in new-born piglets with significant prevalence [50]. In the current study, suckling piglets were examined from the third day of life. Thus, it cannot be ruled out that the unfavourable floor in the farrowing pen may have played a role in the development of the extensive signs identified in the heels, soles and claws. However, the piglets also showed strong signs in their tails, especially at the base, which is difficult to explain by technopathy. Tail bite attacks from other piglets can be excluded during this phase of life, because the piglets were strictly controlled every day. No wounds and marks typical of biting were observed. The considerable influence of sow quality and improved husbandry (in the form of a better water and fibre supply) on the degree and prevalence of the signs also speaks against the exclusive role of unfavourable environmental conditions in the development of the clinical findings. It is conceivable that unfavourable floor conditions in pre-damaged tissues led to more severe injuries, inflammation and necrosis than in intact tissues. However, thus far there has been no research to confirm or refute such a hypothesis. Thus, an important future issue will be to look closely at the interaction between SINS and floor quality. Overall, the current results for the suckling piglets confirm the study by Reiner et al. [51]. The clinical indications for inflammation and necrosis correspond well with the histological findings. Even in animals with macroscopically intact epidermis, we were able to detect considerable prevalence of vasculitis, intima proliferation and thrombosis of the vessels in the tail and ear, especially in suckling piglets. Defects of the epidermis also occurred, but the present study has not clarified whether these were exclusively externally conditioned or whether they were partly caused by the inflammation and necrosis inside the acra. Epidermal damage and granulocytic inflammation increased with age. This effect may be partly due to environmental stressors (noxae), including biting, to which weaners and fattening pigs may have been exposed to an unspecified degree and which may have led to epidermal injuries, separation and bacterial infection. The ears were less affected than the tails, both clinically and histologically. This finding is consistent with the observations in the field and may be attributed to different tissue sensitivities or different stressors.

Some of the animals had intact, some had defective epidermis on the examined body part. For lesions of the epidermis, purulent inflammation predominated in suckling piglets, whereas in cases where the epidermis was intact, lymphoplasma cellular inflammation as a more prolonged to chronic form of a mononuclear inflammation without secondary bacterial infection was the most frequent finding. This suggests ongoing local inflammatory processes in the dermis of these regions. Older piglets and fattening pigs were less affected.

In accordance with the second hypothesis, clinical signs for SINS also occurred in weaners and fatteners. The signs at the base of the tail, teats, navel, face, coronary band and claw wall decreased significantly with age. In weaners, maximum prevalence was found in the tail, ears and sole area, while in fatteners the tail, ears and soles were also frequently affected. Histologically, the effects found in the suckling piglets were superimposed by damage and secondary infections from the environment in the older animals, but were still visible in parts.

The third hypothesis, that the clinical SINS score could be improved through improved husbandry (e.g., better supply of water and fibre), was also confirmed. This hypothesis is based on experience from working farms and published results on intestinal health: Microbiota dysbiosis and intestinal barrier impairment are associated with the development of a number of chronic inflammatory disorders and systemic diseases in humans (for a review, see [1, 68]) and the pathogenic involvement of endotoxins has been reviewed [4, 5, 9, 17]. Thanks to continuous monitoring by the herd health service of Baden-Wurttemberg, specific infectious intestinal diseases can be excluded. The mycotoxin loads of the sows were also controlled. Maximum DON and ZEN levels in feed of 0.3 mg/kg and 0.045 mg/Kg, respectively, were lower than the corresponding guidance values for critical dietary DON and ZEN concentrations of 0.9 mg DON/kg and 0.25 mg ZEN/kg [16]. These typical diet background levels were mirrored by low content in the blood and bile of the piglets. Besides DON and ZEN, one feed sample reached a concentration of 0.17 mg total EA/kg feed, which is approximately half the critical diet concentration of 0.33 mg/kg for primiparous sows as recommended by Kopinski et al. [28] based on the impairment of prolactin concentration in blood. Due to the fact that this critical diet level applies for total EA from *Claviceps africana*, the German recommendation is that critical diet concentrations of total EA should not exceed 0.03 mg/kg, employing an uncertainty factor of 10 to account for the differences in EA pattern between *C. africana* and *C. purpurea*, the predominant ergot form in Europe (https://www.bmel.de/SharedDocs/Downloads/Tier/Futtermittel/Stellungnahme%20der%20Arbeitsgruppe%20%E2%80%9ECarry%20over%22%20zu%20Ergotalkaloiden.pdf?__blob=publicationFile). Thus, the involvement of mycotoxins cannot be completely excluded under the given field conditions. Improving the supply of water and fibre led to a considerable reduction in SINS scores across all age groups. Unfortunately, for experimental reasons, it was not possible to examine the animals in standard husbandry conditions at the same time as those in improved husbandry conditions. This means that the substantial improvement in SINS scores cannot necessarily be attributed solely to the improvement in the supply of water and crude fibre. Unsystematic effects that may have affected both groups of animals to different degrees cannot be definitively excluded. However, no such effects were obvious during the study. The consistency of the improvement across all age groups after the improved administration of water and crude fibre also speaks in favour of the fundamentally beneficial effects of both measures. Improving water and raw fibre supply is generally expected to have a high impact on intestinal health and on the improvement of animal welfare in pigs [31, 33, 36, 62]. Insufficient water intake pre- and post-partum have been proposed as risk factors in, inter alia, postpartum dysgalactia syndrome (PPDS), a globally distributed disease in puerperal sows. The resulting constipation can lead to strong bacterial growth and flooding of endotoxins [20, 21, 49]. Both can be worsened by low fibre [49, 55, 56]. Increasing fibre is “probably the most-cited factor to reduce PPDS” [32]. The role of coprostasis and water supply in PPDS is well described [3, 19] and both have been identified as risk factors for PPDS [24].

As expected, in the current study coprostasis occurred exclusively under standard husbandry conditions, i.e., without improved water and raw fibre supply. Additionally, the coprostasis observed during the first days after birth occurred more frequently in sows with poorer quality teats and claws. Coprostasis had significant consequences for the SINS score of suckling piglets and even more so for weaners. The sow coprostasis factor explained 69% of the variance in the SINS score in weaners, which confirms our fourth hypothesis. Using this fact, an easily to apply prognostic characteristic for SINS can be determined in the early puerperal sow.

In addition to the intestine, bacterial colonization of the endometrium, the mamma and the urinary bladder were identified as further sources for bacterial degradation products, causally for PPDS [3, 19, 22, 26]. Hirsch et al. [21] point to the involvement of injuries and fissures in the teats and claws and identify laminitis as a further source of bacterial products in the syndrome. This led to a further hypothesis in the current study: that sows with intact teats and claws would have a lower burden on their piglets than sows with injuries and lesions in their claws and teats. The 123 sows available in this study showed a considerable variation in teat and claw quality. Thus, 20 sows with extremely favourable and unfavourable values were available for the experiment and to be examined simultaneously. The high degree of differentiation between both sow groups allowed us to demonstrate a considerable effect of sow quality on the degree of SINS in the offspring, at least under standard husbandry conditions. However, the effect was at most 38% of the effect achieved by improving husbandry (water and fibre). Although the sows were assessed on the 50th day of gestation and divided into the experimental groups, the greatest effects on SINS in their offspring occurred in the weaners. This result suggests that the claw and teat traits must have had a lasting effect on the piglets.

In order to determine what proportion of epidermal defects is caused by internal (inflammation of the blood vessels) versus external effects (injuries), individual animal experiments would have to be carried out under laboratory conditions. It is impossible to answer this question within the scope of a field trial, because with increasing age an interaction between both kinds of effect would always have to be assumed. However, discovering this should be high priority, because it is not possible to optimize animal welfare in pigs if only bites and technopathies are controlled, but some of the causes are based on impaired intestinal health and genetic effects (i.e., ultimately endogenous effects). Our results support this view in three respects: i) the shorter the exposure of piglets to the environment, the clearer the SINS problem; ii) inflammatory changes in the vascular area, a major aspect of the hypothetical pathogenesis, could be demonstrated by histological examinations, even in tissues with intact epidermis; iii) coprostasis in the sow showed highly significant effects on the SINS problem not only in suckling piglets but also in weaners and even in fattening pigs, in each case with exactly the same housing environment and feeding.

The hypothesis that inflammation and necrosis in such different organ systems could be explained solely by aggression, behavioural disorders and technopathies must be discarded, not least because of the high degrees of variance of these characteristics explained by coprostasis in the sow and against the background of the explanatory models shown. The same applies, albeit with experimental limitations, to the effects of improved husbandry, in which no changes were made to the floor or other elements of husbandry.

Lighter tail lesions due to interaction with other pigs cannot be completely ruled out in weaners and fatteners because the animals could not be continuously monitored. However, all animals were checked daily and moderate or even severe bite injuries would not have remained hidden. Studies on tail biting in pigs show that water and raw fibre supply as well as intestinal health can play a decisive role [10, 11, 13, 31, 57, 67] and that LPS can increase animals’ aggression towards each other [61]. Future studies will be reserved for identifying the points of intersection between the two problem areas.

The results shown are probably not representative for the pig as a species. From our own investigations ([50, 51]; unpublished data), we know that there are sometimes considerable differences in breed and line. Offspring of Pietrain boars seem to be much more sensitive than the offspring of some other breeds. This question was not the subject of the current study; however, it was decisive in the selection of the experimental animals. In particular, it seemed important to limit the study to a uniform crossbreeding combination with suspected SINS sensitivity to avoid complicating bias from different genetic lines with unknown predisposition. At the same time, the scope of the investigation did not allow the simultaneous testing of different genetic lines. This factor must be considered when conducting similar studies. In line with the hypothetical pathogenesis of SINS outlined above, feeding and husbandry effects with an impact on the pathogenesis should also have a modifying effect on the outcome of SINS studies. However, this is the chance to screen for genetic and husbandry conditions that may have a beneficial effect on inflammation and necrosis and improve welfare in pigs.

## Conclusion

Swine inflammation and necrosis syndrome affects several different bodily regions in pigs such as the tail, ears, teats, coronary bands, heels and claws. The variance in the severity of the syndrome is explained to a large extent by husbandry, in particular by an insufficient supply of hygienically impeccable water and additional raw fibre, as well as by the presence of coprostasis in the mother sow. There is also a significant correlation between the sow teat and claw quality and the SINS score of their offspring. Inflammation and necrosis lead to pain, suffering and damage in the affected animals. In order to exploit every opportunity for improving animal welfare, the results of the present study should be considered in animal husbandry practice and the underlying mechanisms should be elaborated in detail.

## Data Availability

The datasets used and analysed during the current study are available from the corresponding author on reasonable request.
